# Trainee colonoscopists fulfil quality standards for the detection of adenomatous polyps

**DOI:** 10.1186/s12909-015-0312-7

**Published:** 2015-02-27

**Authors:** Peter Klare, Stefan Ascher, Stefan Wagenpfeil, Daniel Rapp, Monther Bajbouj, Bruno Neu, Roland M Schmid, Stefan von Delius

**Affiliations:** 1 II. Medizinische Klinik, Klinikum rechts der Isar der Technischen Universität, Ismaninger Str. 22, 81675 München, Germany; 2 Institut für Medizinische Biometrie, Epidemiologie und Medizinische Informatik, Medizinische Fakultät der Universität des Saarlands, Kirrberger Straße 100, 66424 Homburg/Saar, Germany

**Keywords:** Adenoma, Colorectal, Colonoscopy, Education, Screening, Trainee

## Abstract

**Background:**

The detection of adenomatous lesions is a major indicator for quality and competence in colonoscopy. Little is known about adenoma detection rates (ADR) of endoscopy trainees. The aim of our study was to investigate the performance of trainee colonoscopists in detecting adenomas and to depict the shape of adenoma detection learning curves during apprenticeship.

**Methods:**

We retrospectively investigated a prospectively maintained database of a single tertiary referral center to reveal colonoscopies performed by trainee endoscopists during 2001 and 2013. Colonoscopy reports were chronologically retrieved and separately analyzed for each trainee. Using cumulative curves, courses of trainee’s Adenoma detection rates (ADR) during apprenticeship were displayed. Additionally, procedural data including cecal intubation rate and occurrence of complications were assessed.

**Results:**

We retrospectively analyzed 4354 colonoscopies conducted by 10 trainee endoscopists (TE). A median number of 371 investigations were performed by each apprentice. Group ADR was 23%. No significant difference between aggregated ADRs at the beginning (23%) and at the end (22%) of apprenticeship could be determined (p = 0.70). However, individual learning curves showed considerable different slopes. Personal ADR values ranged between 17% and 31%. Overall cecum intubation rate was 99.0 %. Complication rates were low and fulfilled quality requirements recommended in guidelines.

**Conclusion:**

From the beginning of education, trainee colonoscopists are capable to provide high-quality investigations considering the detection of adenomas as a benchmark quality indicator. Nevertheless, performance differs markedly between investigators. Therefore, individual detection rates should be reviewed regularly to reveal further need for training.

## Background

Colonoscopy is an endoscopic examination which requires both technical and cognitive skills. Teaching and learning colonoscopy still mainly occurs in form of an apprenticeship model which means that beginners observe and imitate skills from expert colonoscopists [1]. Although recommendations exist in order to guide teachers and trainees through apprenticeship [2-4] a precisely structured curriculum is lacking. With respect to the technical part, successful learning has been defined as the ability to intubate the cecum sufficiently [1,5-7]. In contrast, recent colonoscopy training studies have tried to establish score systems including a multitude of assessment tools in order to display skills better and to give a more realistic picture of competence in performing colonoscopy [8-12]. Most importantly, competent endoscopists must be able to detect and remove adenomatous polyps. The detection of adenomas is broadly accepted as a major quality measure in colonoscopy [13,14]. Interestingly, ADRs strongly vary between endoscopists, a fact which has not been explained unambiguously [14-17]. Even less is known about the performance of trainees regarding the skill of detecting adenomatous lesions. It is not clear whether detection rates change during apprenticeship and if they do, how such learning curves would be shaped. Furthermore, though minimum investigation numbers are established for colonoscopy as a promise of certification, these numbers have not been sufficiently related to ADRs until now. To illuminate these issues we retrospectively analyzed the performance of 10 trainee colonoscopists with respect to ADRs.

## Methods

### Study design & data collection

Our sequential and prospectively maintained institutional database was retrospectively searched to identify all colonoscopy reports that were generated by 10 trainee endoscopists (U1 – U10) within a 12 year timeframe (2001 – 2013) at our institution. All fellows being inexperienced in lower gastrointestinal endoscopy were included. Performance of any colonoscopy procedure at other centers before served as an exclusion criterion. We considered colonoscopies which were actually conducted by trainee endoscopists (TE). Each series started with the first investigation conducted independently by the respective trainee. Procedures which were passively observed by trainees during former didactic sessions (see section “Educational Standards”) were not included in the analysis. Sigmoidoscopies and investigations in which the maximum insertion was intended to be lower than the cecum were not considered. We furthermore excluded incomplete investigations due to stenosis as well as colonoscopies which had to be terminated prematurely due to insufficient bowel cleansing.

Regarding ADR calculations we intended to avoid distortion by cases in which indication for colonoscopy were namely polypectomy, surveillance for inflammatory bowel disease or polyposis syndromes. These investigations were therefore considered only for calculation of the amount of investigations performed by trainees (“hands-on experience”). Adenoma detection was not considered in these cases. Instead, a fictive ADR value was assigned to these colonoscopies consisting of the investigators own detection rate calculated up to that point in time. By that, we prevented any impact of the named cases on the investigators ADR but considered the investigations in the cumulative presentation in order to illustrate hands-on learning effects.

We recorded number, localization and size of detected polyps and tumors. Lesions detected in an area beginning with the cecum up to the splenic flexure were assigned to the right (proximal) colon. All polyps further down were called left-sided or distal lesions. According to histopathological findings lesions were divided in the following categories: no pathology, hyperplastic polyp, adenoma (tubular, villous, serrated) and carcinoma. We defined adenomas which presented villous histology or were larger than 10 mm size as “advanced lesions”. Adenocarcinomas were also considered advanced lesions. In cases where no polypectomy was conducted or histopathological data was not available, lesions were not allocated to one of the above mentioned categories.

Colonoscopy reports were also evaluated with regard to procedural measurements, particularly date of investigation, indication for procedure, bowel preparation, amount of sedatives used and success of cecal intubation. Reported complications during investigation were divided in minor complications, bleeding, perforation and severe respiratory insufficiency (need for intubation or mask ventilation).

The study was approved by the Ethics Committee of the Technical University of Munich (project number: 5671/13). In addition we registered the trial at the ClinicalTrials.gov database (ID: NCT01786213). Written informed consent was not obtained from patients as the analysis of data was conducted retrospectively.

### Endoscopy procedure and educational standards

All gastroenterology fellows had reached competency in upper endoscopy before the beginning of colonoscopy education. The mean number of gastroscopies performed was 401 ± 193. No fellow had performed any colonoscopy procedure at other centers before the beginning of colonoscopy training in our department. As part of the colonoscopy training regime, TEs received didactic sessions in which fellows observed senior endoscopists at work prior to the beginning of hands-on training. Trainees received a mean number of 8.5 ± 5.1 didactic sessions prior to the conduction of the first colonoscopy.

TEs were supervised closely by experienced endoscopists during the first 50 consecutive investigations. Afterwards, experienced endoscopists did not attend the examination room any more but were available on call. This means that in case of difficulties reaching the cecum, or interventions endoscopes were taken over by a routinier but instruments were given back to the apprentice afterwards.

Bowel cleansing regime usually consisted of at least 2 liters macrogole administered as a split dose. We used propofol alone or a combination of propofol and midazolam as sedation regime. All procedures were done with standard white light video-colonoscopes. Regarding colonoscopy techniques the “one-man-only” method was used by default. When polyps were found lesion size was estimated by comparison with the biopsy forceps or snare. In accordance with current guidelines [18] resections were done by biopsy forceps, if lesions were equal to or less than 5 mm. Otherwise, snare resection was performed. Identification of the cecum was documented by taking pictures of the ileoceacal valve or the appendix orifice.

### Descriptive and inferential statistics

Each series started with the first investigation carried out by the respective TE. Colonoscopy reports were then brought into a chronological order separated for each TE. Adenoma detection rate (defined as number of colonoscopies in which at least one adenomatous lesion was found divided by total amount of investigations) was calculated for each TE at intervals of 25 consecutive records. Group adenoma detection rates were displayed block by block from 25 up to 250 investigations and cumulative curves were computed. The same applied for investigator-specific ADRs. Since Trainees conducted different numbers of colonoscopies during the overall period, we also calculated ADR values with respect to the final extent of investigations.

Descriptive statistics is due to means and ranges for quantitative variables as well as relative and absolute frequencies for qualitative variables. We computed linear trend regression lines to show the influence of numbers of colonoscopies performed on ADR on the whole and separately for each of the investigators. Statistical analysis was performed with Microsoft EXCEL 2010 and IBM SPSS version 21.

## Results

### Patient characteristics and procedural aspects

Database query revealed a total of 4682 colonoscopies carried out by the 10 TEs in the respective time frame between 2001 and 2013. We excluded 328 cases due to inappropriate investigations as defined above. Of these 128 were terminated prematurely due to poor bowel preparation. Another 178 had stenosis or maximal insertion a priori was intended to be lower than the cecum. Thus, a total of 4354 reports were analyzed.

Patients had a mean age of 59 years (range: 18 – 98). Population consisted of 2385 males (54.8%) and 2082 females (45.2%). Screening and surveillance after former polypectomy comprised for 970 (22.3%) indications. Further indications were bleeding/anemia (1196, 27.5%), diarrhea or obstipation (436, 10.0%) abdominal pain (398, 9.1%), suspected tumor (485, 11.1%) and others (869, 20.0%) (Table 1).

**Table 1:** **Patient characteristics and procedural measures of 4354 cases**

Patients characteristics	
Age (y)	59 (18 - 98)
Gender: Male/Female	2385/1969 (55%/45%)
**Procedural measurements**	
Indication:	
*Screening*	970 (22.3%)
* Anemia*	1196 (27.5%)
* Diarrhea/Obtipation*	436 (10.0%)
* Abdominal discomfort*	398 (9.1%)
* Suspected tumor*	485 (11.1%)
* Others*	869 (20.0%)
Using Midazolam	1768 (40.6%)
Midazolam dose (mg, mean)	2.5 mg (range: 0.5 - 7.5)
Using Propofole	3550 (81.5%)
Propofole dose (mg, mean)	192 mg (range: 20 to 1100)
Bowel cleanness (light to moderate remnants/excellent)	1170/3184 (26.9%/73.1%)
Complications overall	45 (1.0%)
* Minor complications*	27 (0.6%)
* Bleeding*	13 (0.3%)
* Perforation*	4 (0.9‰)
* Severe respiratory insuffiency*	1 (0.2‰)

Numbers are mean values (range: minimum – maximum) or frequencies (percentages).

Most patients (3675, 84.4%) received sedatives during endoscopy. Propofol was used in 3550 (81.5%) cases with an average dose of 192 mg (range: 20 to 1100 mg). A total of 1768 patients (40.6%) received midazolam. In 1631 cases a standard dosing of 2.5 mg midazolame was administered per session whereas in 137 cases lower or higher dosages were used.

Overall cecal intubation rate was 99%. As stated above experienced endoscopists were available on call in case where trainees had difficulties reaching the cecum. Against the background of this setting no personal intubation rates were calculated for trainees. Regarding bowel cleanness investigators described mild to moderate fecal remnants in 26.9%. Based on 45 documented complications we calculated a total complication rate of 1.0%. Minor complications comprised for the majority of these events (27/45, 60.0%). No fatal complications occurred. In one single case, intubation had to be carried out due to aspiration accompanied with severe respiratory insufficiency. Severe bleeding with the need for a second look and endoscopic hemostasis was noticed in 13 cases. In no case transmission to surgery was necessary due to bleeding. Perforation occurred in four cases resulting in a perforation rate of 0.9 events per thousand investigations. In two cases surgery had to be carried out due to perforation (0.5 per mill). One patient was treated conservatively whereas in another case perforation was managed endoscopically using an over the scope clip. Patient characteristics, indication and procedural aspects are shown in Table 1.

### Trainee performance in adenoma detection

All 10 TEs were men. Each TE conducted a median number of 371 investigations (range 237 – 999) during the analyzed time span. Median number of colonoscopies per year was 127. For completion of the first 250 consecutive investigations TE required a median time span of 21 month. We analyzed 3782 cases for the calculation of adenoma detection rates. A median number of 313 colonoscopies per trainee were investigated (range: 213 – 863) regarding this matter. A total of 3064 lesions were detected including 1537 adenomas. Out of these 24.7% (379 lesions) were classified as advanced adenomas. We detected 120 adenocarcinomas representing an overall rate of 3.0%. Overall, polyp and adenoma detection rates were 42% and 23% respectively (Table 2).

**Table 2:** **Lesions detected by trainee endoscopists during 3782 colonoscopies**

Lesion	Total/Detection rate
Lesions total (Polyps, Carcinomas)	3064
Polyps	2944
*Adenomas*	1537
among them: *Advanced Adenomas*	379
* Hyperplastic Polyps*	632
* No polyp/normal mucosa (according to pathological report)*	503
* Unresected polyps:*	
* due to coagulopathy/blood-thinning medications*	175
* due to insufficient bowel preparation*	32
*Polyps being lost after resection*	31
Carcinomas	120
Polyp detection rate*	42%
Adenoma detection rate*	23%
Cases with at least 1 Adenoma	862
Cases with 2 or more Adenomas	339
Advanced Adenoma detection rate*	7%
Cases with at least 1 Advanced Adenoma	281
Cases with 2 or more Advanced Adenomas	54

Values are presented as n (%).

*Detection rates were defined as number of colonoscopies in which one or more lesion was found divided by the number of colonoscopies performed.

**Figure 1:**
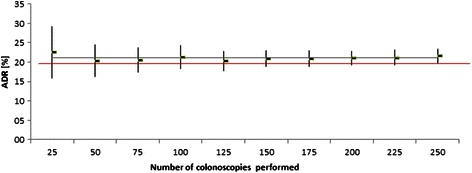
**Green points show ADR values (mean) of ten TE during apprenticeship.** Vertical bars signify 95% Confidence Intervals. ADRs were calculated in blocks of 25 consecutive investigations. Linear regression is displayed by the blue line (red line: recommended minimal requirement (20%)).

**Figure 2:**
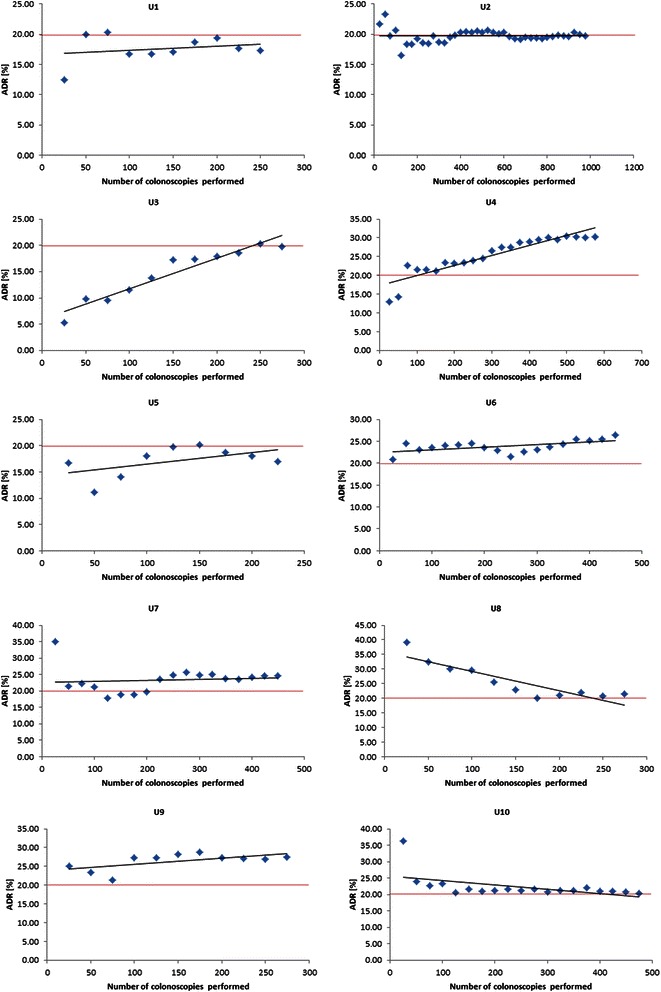
**Points represent ADR values calculated in blocks of 25 consecutive investigations.** Dark shaded line shows linear regression. Red line: recommended minimal requirement of 20%.

Group ADR calculations after 25 and 50 consecutive investigations revealed mean values of 22.6% and 20.4% and therefore already exceeded the 20% mark. The trend of global adenoma detection over time is shown in Figure 1. Confidence intervals (95% CI) of computed group ADR values (vertical bars in Figure 1) were higher at the beginning of apprenticeship (after 25 or 50 investigations) and subsided towards the end of the observation period. Comparison of ADR values at the points 25 vs. 250 and 50 vs. 250 colonoscopies revealed no significant difference (p = 0.70 and p = 0.92, respectively). Trainee’s personal adenoma detection rates ranged from 17 to 31% (Table 3). Figure 2 shows individual learning curves of all 10 trainees. The five trainees with the highest ADR values also revealed the highest Polyp detection rates (PDR) and were most successful in the detection of >1 polyp per session (Table 4). Median polyp and adenoma size measured by the ten trainees did not differ markedly (Table 4).

**Table 3:** **Number of colonoscopies and ADRs of 10 Trainee Endoscopists (U1-U10) during apprenticeship**

TE	Colonoscopies (total)	Colonoscopies (valid for ADR calculations)	ADR (%)
**U1**	271	240	16.7
**U2**	999	863	19.8
**U3**	280	252	19.8
**U4**	596	512	30.7
**U5**	237	213	17.4
**U6**	454	397	26.7
**U7**	467	361	24.9
**U8**	288	251	21.5
**U9**	286	264	26.5
**U10**	476	429	20.3

Total observation time span: 2001 - 2013. Discrepancy between total number of colonoscopies and investigations valid for ADR calculaction derives from the exlusion of cases in which polyp resection, FAP and IBD were indications for procedure. ADR: number of colonoscopies in which at least one adenomatous lesion was found devided by total amount of investigations.

**Table 4:** **Individual Polyp and Adenoma detection rates of 10 Trainee Endoscopists (U1-U10)**

TE	Procedures with ≥ 1 Polyp (PDR)	Procedures with ≥ 2 Polyps (Percentage)	Procedures with ≥ 1 Adenoma (ADR)	Procedures with ≥ 2 Adenomas (Percentage)	Median Polyp size (min – max)	Median Adenoma size (min – max)	Polyps detected (total)	Adenomas detected (total)
**U1**	75 (31.3%)	32 (13.3%)	40 (16.7%)	14 (5.8%)	3 (2 - 20)	4 (2 - 20)	122	76
**U2**	323 (37.4%)	137 (15.9%)	171 (19.8%)	69 (8.0%)	4 (1 – 35)	5 (2 – 35)	588	312
**U3**	81 (32.1%)	31 (12.3%)	50 (19.8%)	14 (5.6%)	3 (2 – 30)	5 (2 – 21)	127	70
**U4**	267 (52.1%)	154 (30.1%)	157 (30.7%)	74 (14.5%)	3 (1 – 35)	4 (1 – 35)	706	318
**U5**	77 (36.2%)	28 (13.1%)	37 (17.4%)	13 (6.1%)	3 (2 – 20)	4 (2 – 8)	141	64
**U6**	199 (50.1%)	87 (21.9%)	106 (26.7%)	38 (9.6%)	3 (2 – 25)	3 (2 – 20)	392	173
**U7**	151 (41.8%)	69 (19.1%)	90 (24.9%)	37 (10.2%)	4 (2 – 25)	4 (2 – 25)	291	167
**U8**	109 (43.4%)	46 (18.3%)	54 (21.5%)	22 (8.8%)	2 (2 – 15)	4 (2 – 15)	194	94
**U9**	134 (50.8%)	60 (22.7%)	70 (26.5%)	31 (11.7)	3 (2 – 17)	5 (2 – 17)	237	131
**U10**	163 (38.0%)	63 (14.7%)	87 (20.3%)	27 (6.3%)	2 (2 – 20)	4 (2 – 20)	266	132

Numbers are frequencies (percentages) or median (minimum-maximum); Polyp and Adenoma size in millimeter.

## Discussion

In this study we evaluated the performance of trainee colonoscopists with regard to group and individual adenoma detection rates during apprenticeship. Motor and cognitive skills has been demonstrated to improve depending on the number of colonoscopies performed [9,12]. We therefore assumed to find a likewise trend concerning the detection of adenomas since controlling the endoscope and identifying colonic lesion are indispensable skills for adenoma detection. Interestingly, in our study trainees fulfilled quality standards from the very beginning. Trainees averagely reached a 23% ADR after only 25 investigations and no skip below the 20% mark was noticed at any point during apprenticeship. Trainees were supervised closely by experienced endoscopists during the first 50 investigations. Thus, early ADR values might represent merged parameters valid for both trainee and trainer. Therefore, the nature of training has to be kept in mind when interpreting these results. Following this initial period experienced endoscopists were only available on call in case of technical difficulties. At least from this point on, ADR values are capable to reflect trainees’ performance. Based upon our data we conclude that the risk of missing adenomas due to deficient competence of trainee endoscopists is rather low. Nevertheless, in one back-to-back colonoscopy study with 147 tandem investigations authors found that poor experience was a predictor for increased adenoma miss rates [19]. As stated above, the fact that trainees were supervised at the very beginning of education may have resulted in an overestimation of ADR values.

Some studies have investigated detection rates of apprentices before. Comparing initial and final ADR values of trainees, no significant differences could be verified [20-22]. In one recent prospective study Gromski surveyed 4 trainee endoscopists for a total of 1210 investigations and calculated an overall ADR of 22% [22]. In accordance with our data no significant difference between ADR values after 50 and 200 consecutive colonoscopies was reported. However, Gromski and colleagues also stated no significant difference between trainee’s personal ADRs. In the present analysis individual ADR learning curves showed considerable differences. Personal detection rates ranged between 17% and 31%. Trainees with the best ADRs also revealed the highest PDRs and were most successful in detecting more than 1 polyp per session. Interestingly, while most ADR regression lines showed rather flat gradients, some candidates presented both increasing as well as decreasing gradients (Figure 2). A downward- sloping curve might to some extent reflect successful adenoma detection at the beginning caused by the attendance of experienced trainers. This explanation would entail that some trainees, due to a personal inability to detect sufficient numbers, are in need for a greater supervision during apprenticeship. A recent retrospective survey investigating the performance of gastroenterology and surgical trainee colonoscopists provided insight into the variability of trainee’s ADRs. Both, gastroenterological and surgical trainees reached ADR values below 20%. Furthermore, gastroenterology novices performed significantly better than surgical colleagues [23]. Due to these results authors claimed the need for a common curriculum containing structured and comparable training standards [23].

The extent or time of observation is a crucial point when measuring the competence of detecting adenoma during apprenticeship. How many investigations should be surveyed within a study measuring adenoma detection rates of trainee colonoscopists? As stated above, exact numbers of procedures, that must be performed for reaching competency in colonoscopy are still lacking. The European Society of Gastrointestinal Endoscopy solely advocates the establishment of minimum annual and lifetime benchmarks for investigators performing screening colonoscopies [24]. Gastroenterological societies in the United States recommend an unadjusted cecum intubation rate of at least 90% and the performance of at least 140 investigations [13,25]. The latter requirement has recently been discussed controversially. Though some data seem to confirm the named benchmark number [22] other studies have revealed much higher number of colonoscopies to ensure sufficient quality standards. Sedlack showed that in average 275 colonoscopies were required until an 85% cecal intubation rate was reached [10]. Spier supervised eleven trainee colonoscopists during an 18 month apprenticeship and found that none of them reached cecal intubation defined competence after 140 colonoscopies [21]. These findings, together with the insight that a mere surrogate parameter may not be sufficient to evaluate competence, have enforced the American Society of Gastrointestinal Endoscopy (ASGE) to recommend a new colonoscopy skill assessment tool for colonoscopy [26]. The Assessment of Competency in Endoscopy (ACE) form consists of a further development of the Mayo Colonoscopy Skill Assessment Tool (MCSAT) and takes into account both practical and cognitive measures. Furthermore, the ACE form also considers histopathological findings of lesions detected by learners [26]. Right now the use of the ACE tool is at its very beginning. Future data will have to proof weather the use of such tools are capable to answer the question at which point learning comes to an end and which number of colonoscopies may serve as a valid benchmark for competence.

In our study we decided to evaluate an amount of 250 consecutive investigations performed by 10 trainees each. As discussed above we are not able to prove this amount to be sufficient for the investigated problem but at least it is in accordance with current standards. Some additional parameters suggested satisfactory attainment of competence. This is supported by low complication rates which were in accordance with quality standards recommended by guidelines [13,24]. Overall, we believe that the amount of colonoscopies covered a long enough time span. As a consequence, our data should also provide valid information about trainee’s capability in detecting adenomas. Nevertheless, we observed high discrepancies between individual performances. One interpretation of this fact could be that some candidates have not reached the end of their own individual learning curve within our setting and that data analysis would need more cases to achieve a correct picture. On the other side it would be conceivable that personal detection rates are not completely dependent on the amount of training but somehow are to consider as an investigator–specific feature. Anyway, our data should enforce us to implement a regular and continuous evaluation of adenoma detection rates during education in colonoscopy. By that, trainees and supervisors will have the opportunity to discover individual deficits and to use this awareness for adaption of training.

### Limitations

First, due to the retrospective nature of the study results may be distorted or incomplete. Prospective studies should be performed to confirm our results. Second we were faced with a mixed patient population undergoing colonoscopy for a multitude of reasons. Quality standards for colonoscopy such as a standardized bowel cleansing, adenoma detection and cecal intubation rates have been established for screening colonoscopies of asymptomatic adults in the narrower sense. Third, colonoscopies took place at a single tertiary referral center which might explain the high proportion of advanced lesions and carcinoma which were detected.

Finally, our presented results are based on center specific apprenticeship modalities which are preliminarily characterized by a close supervision of trainees by experienced colonscopists during the first 50 investigations. This fact has to be considered as one major limitation when discussing trainees’ early ADR values.

## Conclusions

In summary, our data support the assumption that trainee endoscopists are able to provide high quality investigations during apprenticeship. On average ADR of trainees was 23%. During the whole period of observation ADR did not fall below a 20% mark. Other surrogate parameters for high quality investigations like sufficient cecal intubation and low complication rates were also fulfilled. Individual ADR learning curves showed noticeable differences. Therefore, we conclude that a regular and continuous evaluation of adenoma detection rates should be an integral part of a colonoscopy curriculum.
